# Epigenetics for Crop Improvement in Times of Global Change

**DOI:** 10.3390/biology10080766

**Published:** 2021-08-11

**Authors:** Ioanna Kakoulidou, Evangelia V. Avramidou, Miroslav Baránek, Sophie Brunel-Muguet, Sara Farrona, Frank Johannes, Eirini Kaiserli, Michal Lieberman-Lazarovich, Federico Martinelli, Velimir Mladenov, Pilar S. Testillano, Valya Vassileva, Stéphane Maury

**Affiliations:** 1 Department of Molecular Life Sciences, Technical University of Munich, Liesel-Beckmann-Str. 2, 85354 Freising, Germany; i.kakoulidou@tum.de (I.K.); frank@johanneslab.org (F.J.); 2 Laboratory of Forest Genetics and Biotechnology, Institute of Mediterranean Forest Ecosystems, Hellenic Agricultural Organization-Dimitra (ELGO-DIMITRA), 11528 Athens, Greece; avramidou@fria.gr; 3 Faculty of Horticulture, Mendeleum—Institute of Genetics, Mendel University in Brno, Valtická 334, 69144 Lednice, Czech Republic; baranek@mendelu.cz; 4 UMR 950 Ecophysiologie Végétale, Agronomie et Nutritions N, C, S, UNICAEN, INRAE, Normandie Université, CEDEX, F-14032 Caen, France; sophie.brunel-muguet@inrae.fr; 5 Plant and AgriBiosciences Centre, Ryan Institute, National University of Ireland (NUI) Galway, H91 TK33 Galway, Ireland; sara.farrona@nuigalway.ie; 6 Institute for Advanced Study, Technical University of Munich, Lichtenberg Str. 2a, 85748 Garching, Germany; 7 Institute of Molecular, Cell and Systems Biology, College of Medical, Veterinary and Life Sciences, Bower Building, University of Glasgow, Glasgow G12 8QQ, UK; eirini.kaiserli@glasgow.ac.uk; 8 Institute of Plant Sciences, Agricultural Research Organization, Volcani Center, Rishon LeZion 7505101, Israel; michall@volcani.agri.gov.il; 9 Department of Biology, University of Florence, 50019 Sesto Fiorentino, Italy; federico.martinelli@unifi.it; 10 Faculty of Agriculture, University of Novi Sad, Sq. Dositeja Obradovića 8, 21000 Novi Sad, Serbia; velimir.mladenov@polj.edu.rs; 11 Pollen Biotechnology of Crop Plants Group, Centro de Investigaciones Biológicas Margarita Salas-(CIB-CSIC), Ramiro Maeztu 9, 28040 Madrid, Spain; testillano@cib.csic.es; 12 Department of Molecular Biology and Genetics, Institute of Plant Physiology and Genetics, Bulgarian Academy of Sciences, Acad. Georgi Bonchev Str., Bldg. 21, 1113 Sofia, Bulgaria; valyavassileva@bio21.bas.bg; 13 Laboratoire de Biologie des Ligneux et des Grandes Cultures, INRAE, EA1207 USC1328, Université d’Orléans, F-45067 Orléans, France

**Keywords:** breeding, climate change, DNA methylation, epigenomics, memory, plant epigenetics, prediction models, priming

## Abstract

**Simple Summary:**

Research on plant epigenetics aims to understand how endogenous, biotic, and abiotic factors regulate plant development and growth independent of changes in the genome sequence. Often, the epigenetic changes are heritable across generations and modulate plant growth and crop tolerance, particularly in response to environmental stimuli. To take advantage of epigenetic adaptation, recent research has focused on implementing targeted epigenetic diversity to engineer plants that harbour advantageous traits for optimal crop production. Epigenetics has the potential to provide a powerful toolbox for crop breeders; however, most mechanistic studies are based on information from model plant species due to the challenges that arise when working with crops. Here, we summarise the contribution of epigenetics to optimising crop adaptation in response to climate change and overview potential future applications as well as challenges.

**Abstract:**

Epigenetics has emerged as an important research field for crop improvement under the on-going climatic changes. Heritable epigenetic changes can arise independently of DNA sequence alterations and have been associated with altered gene expression and transmitted phenotypic variation. By modulating plant development and physiological responses to environmental conditions, epigenetic diversity—naturally, genetically, chemically, or environmentally induced—can help optimise crop traits in an era challenged by global climate change. Beyond DNA sequence variation, the epigenetic modifications may contribute to breeding by providing useful markers and allowing the use of epigenome diversity to predict plant performance and increase final crop production. Given the difficulties in transferring the knowledge of the epigenetic mechanisms from model plants to crops, various strategies have emerged. Among those strategies are modelling frameworks dedicated to predicting epigenetically controlled-adaptive traits, the use of epigenetics for in vitro regeneration to accelerate crop breeding, and changes of specific epigenetic marks that modulate gene expression of traits of interest. The key challenge that agriculture faces in the 21st century is to increase crop production by speeding up the breeding of resilient crop species. Therefore, epigenetics provides fundamental molecular information with potential direct applications in crop enhancement, tolerance, and adaptation within the context of climate change.

## 1. Introduction

Based on the Paris Agreement in 2015, the United Nations agreed to limit global warming to 2.0 °C with the ambition to attempt to cap warming at 1.5 °C (UNFCCC, 2015). Greenhouse gases (GHGs) released from human activities are universally recognised as the most significant driver of shifts in climate change observed since the mid-20th century (IPCC, 2013). Agriculture, forestry, and other land uses are responsible for almost a quarter of anthropogenic GHG emissions [1], and a reduction in agriculture-derived emissions is essential for limiting global warming [2]. On the other hand, numerous studies have shown that agricultural crop production and food security is one of many sectors already affected by climate change [3,4,5]. Recent reports on the impact of climatic trends on wheat, maize, and barley yields based on new statistical and empirical models revealed a negative response of global yields to increased temperatures [6,7]. Besides global warming, biotic constraints, such as pathogens, pests, and weeds, can be detrimental to crop production worldwide. Numerous studies on different crops, such as soybean [8], have revealed the effect of pathogens, pests, and insects on annual soybean production worldwide, and on wheat and cotton, where pest attacks are responsible for damaging more than 50% or 80% of the annual production [9]. A global strategy to minimise major crop losses by optimising crop protection and resilience in response to biotic and abiotic stresses is essential for safeguarding future food availability [10]. To face these biotic and abiotic constraints, emergence for improved adaptation and selection of superior genotypes through the study of epigenetics will have a significant impact on future crop sustainability. 

In eukaryotes, including plants, genomic DNA is tightly packaged into a dynamic but stable nucleoprotein complex, known as chromatin, whose conformation enables DNA accessibility and controls all DNA-based activities [11]. The primary structural unit of chromatin compaction is the nucleosome, comprising DNA wrapped around a core of eight highly conserved histone molecules. The level of nucleosome occupancy orchestrates chromatin accessibility to the transcriptional machinery and to other regulatory proteins, thus affecting gene availability for transcription. In this context, “epigenetic” can be defined as mitotically and/or meiotically heritable changes in gene function that cannot be explained by changes in DNA sequence. Then, modulators of chromatin compaction are regarded as epigenetic marks, including DNA methylation, histone modifications, chromatin remodelers, and to some extent small RNAs [12]. DNA methylation is defined by the covalent addition of a methyl group (CH3) to the fifth position of a cytosine ring (5 mC) by DNA methyltransferases without altering the DNA sequence [13,14,15]. Histone variants and post-translational modifications (PTMs) [16], such as phosphorylation, acetylation, and methylation, are essential elements of the chromatin signalling pathway. Lastly, RNA molecules are either small RNAs (small interfering RNAs-siRNAs and microRNAs-miRNAs) or long non-coding RNAs (long ncRNAs) and have been demonstrated to contribute to phenotypic changes. All these epigenetic marks are closely linked, acting together to coordinate gene activity at the transcriptional level, and regulate different cellular processes, such as DNA replication and repair, stem cell maintenance, the establishment of cell identity, and tissue and organ development and differentiation, but also responses to environmental stimuli [17]. The main benefit derived from the dynamic nature of epigenetic networks is associated with the stability and reversibility of chromatin modifications. The stability of these changes is essential for maintaining epigenetic memory (somatic memory and inter/transgenerational memory), which allows cells to maintain their identity during plant development and “remember” favourable alterations leading to a selective advantage. The reversible nature of epigenetic changes confers plasticity, enabling differentiated cells to regain totipotency, and adequately respond and adapt to internal and environmental stimuli [18].

The relevance of epigenetic regulation to crop breeding has been demonstrated, for example, by its effect on growth vigour and yield in tomato [19]. Silencing of the *MutS HOMOLOG1* (*MSH1*) gene in tomato using RNAi results in enhanced plant growth and productivity, even in the absence of the transgene. Total fruit weight and number are increased under field conditions. In addition, under high-temperature field conditions, the MSH1-silenced line produced a higher proportion of red ripe fruits, similarly to the FLA8044 heat-tolerant cultivar. These phenotypic changes are linked to DNA methylation, as the methylation inhibitor 5-Azacytidine (5-AzaC) represses the observed phenotypes [19]. In a later study, it was shown that METHYLTRANSFERASE 1 (MET1) and HISTONE DEACETYLASE 6 (HDA6) are essential components of these changes [20]. In *Arabidopsis*, *MSH1* mutants displayed enhanced tolerance to drought and salt stress, and increased susceptibility to freezing temperatures [21]. This example, along with various others (Table 1), highlights the link between epigenetics and multiple important traits, as well as the potential of epigenetics in crop breeding. Additional examples demonstrating the importance of epigenetic regulation of crop resilience and productivity to environmental and endogenous factors are anticipated to be uncovered in coming years.

According to the facts stated above, this review aims to decipher the contribution of epigenetics to crop adaptation in response to climate change, which is already affecting or will have a significant impact on crop production. Here, we present a series of different examples of how epigenetics can be applied to improve crop productivity in the following three sections:

Firstly, we discuss the physiological significance of epigenetic changes regulating all aspects of plant development and responses to adverse environmental conditions in the model plant *Arabidopsis* in relation to phenotypic variation and plant reproduction. Potential applications of our knowledge to economically important crop species towards fulfilling crop improvement requirements in an era of global climate change.

Secondly, we present the current knowledge about epigenetic diversity in the view of different aspects, such as: (a) the naturally occurring epialleles and their connection with important phenological traits, (b) the usage of chemicals and modification of epigenetic diversity, (c) the usage of epimutations as a source of transgenerational inheritance potential of epigenetic marks in crops, (d) environmental conditions which are associated with epigenetic diversity through priming and local adaptation, (e) how clonal propagation is associated with epigenetic diversity/DNA methylation and phenotypic divergence and inheritance, and (f) the significance of epi-biomarkers as a tool for predicting superior hybrid performance or as a diagnostic tool, at early developmental stages, for undesired phenotypes in clonally propagated material with high economic impact.

Finally, an overview of how molecular strategies can be improved and implemented in order to study epigenetics more efficiently and to use epigenetic-based approaches for crop improvement is highlighted in this study. These strategies include: (a) approaches and strategies for dissecting detailed epigenetic mechanisms, (b) efficient correlation between phenotypes and epigenome status through modelling, (c) prediction of epigenetically controlled adaptive traits, and (d) epigenome editing.

## 2. From Epigenetics to Crop Improvement: Lessons from *Arabidopsis* and other Model Plant Species

**Figure 1:**
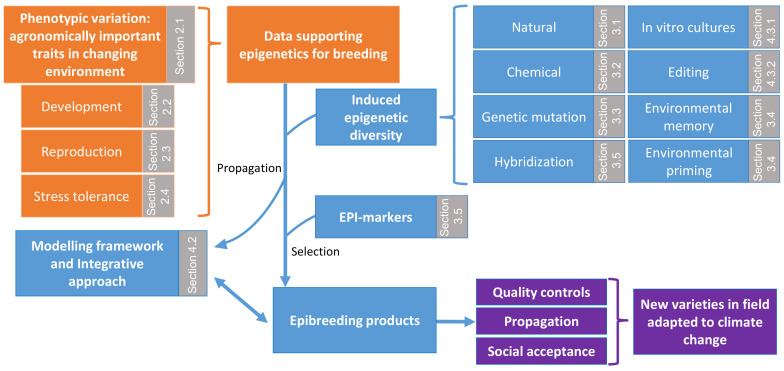
How the development of epigenetic data and tools will lead to epi-bred crops and new varieties in the field adapted to climate change. Section to find each topic in the review is indicated. In brown are indicated data already supporting epigenetics for breeding, in blue the uses of epigenetics for crop improvement (actually under development), and in purple the post-production steps until new varieties in the field (future challenge).

Key advances in our understanding of plant epigenetic machinery derive from species adopted as models in plant biology, such as *Arabidopsis* *thaliana* [22], *Brachypodium distachyon* [23], *Lotus japonicus* [24], and *Medicago truncatula* [25,26]. Other species that represent crop models including *Oryza sativa* [27], *Zea mays* [28,29], *Triticum aestivum* [30], *Glycine* max [31], and *Solanum lycopersicum* [32] provide a source of valuable information about plant-specific epigenetic phenomena and regulators (Table 1). Delivering fundamental knowledge across different aspects of plant adaptability to environmental cues mediated by heritable epigenetic variance will greatly help in improving agriculturally desired developmental or stress-related crop traits (Figure 1).

### 2.1. Epimutations Contribute to Phenotypic Variation in Model Plants

Similarly to genetic mutations, epimutations can occur spontaneously or in an induced manner, and be transmitted to the next generations, producing new epigenetic alleles (epialleles), which provide additional sources of phenotypic diversity [33,34]. Some spontaneous somatic epimutations accumulate during development in an age-related manner [32]. In *A. thaliana*, their number at individual CG dinucleotides is approximately five orders of magnitude greater than the genetic mutation rate [34,35]. In general, changed methylation at a single cytosine residue does not trigger phenotypic alterations in plants. However, methylation changes affecting large genomic regions have been associated with altered gene expression and heritable phenotypic variations. In the model plant *A. thaliana*, such region-level epimutations can occur in CG and non-CG sequence contexts, and have similar rate and spectrum as single cytosine epimutations, which imply their independence from genetic mutations. The region-level changes display strong dependence on chromosomal location, with the highest accumulation in genes and chromosome arms, and the lowest in centromere-specific TEs [36]. Therefore, region-level epimutations provide an additional source of methylome and phenotypic diversity in *A. thaliana* [37]. Interestingly, in the long-lived perennial tree *Populus trichocarpa*, the rates of epimutations per-year are lower than in *A. thaliana*, which has been proposed to be attributed to the limited number of meristematic cell divisions during the tree lifespan as a protection mechanism against environmental mutagens [38]. The same authors conclude that transgenerationally heritable somatic epimutations derive mainly from aberrant DNA methylation maintenance during mitotic cell divisions. On the other hand, a study with two mutation accumulation lines in *A. thaliana* has shown that methylated genome regions are stably inherited across generations, and spontaneous epialleles are rarely observed [39], which is consistent with very recent results on rice hybrids [40]. Spontaneous epimutations that are linked with variations in gene body methylation, mostly in the CG sequence context, frequently do not have functional outputs for the plants [41]. On the other hand, the epi-variants related to transposable elements (TEs) can lead to a loss of DNA methylation in all three sequence contexts, forming potentially stable epialleles that transmit across generations [42]. Thus, stable epi-allelic inheritance tends to rely on the total loss of DNA methylation. Interestingly, Wendte [43] has shown another mechanism that includes initiation of gene body methylation and induction of genic CG epimutations by CHROMOMETHYLASE 3 (CMT3), which can be maintained over generational time.

Epimutations can arise as a consequence of environmental fluctuations, and then can be transmitted to the progeny. Transgenerational epiallelic stability has been observed in a single trait linked to seed dormancy upon drought exposure of *Arabidopsis* [44]. Similarly, multi-generational rice drought treatment resulted in epimutations maintaining changed methylation status in the next generations, as many of them are detected in loci associated with drought resistance [45]. Some of these epimutations may have adaptive potential to drought stress, and could be considered as epigenetic markers for predicting rice drought response. Salinity stress has been reported to induce demethylation of certain regions in the *Arabidopsis* genome, as part of salt-induced responses that are transmitted to their progeny through the female germline, and then lost when this stress no longer exists [46]. Ferreira [47] identified a series of differentially methylated regions (DMRs) in salt-exposed plants, and has noted a general tendency of losing methylation in these regions. The progeny of salt-exposed plants is pre-adapted, but this adaptive response is repressed when RNA-directed DNA methylation (RdDM) or active DNA demethylation pathways are impaired. Therefore, the identified salt-induced variations are primarily involved in intergenerational stress memory. Although long-term epigenetic memory in response to UV light exposure also showed a decreased strength over several generations, some transgenerational epimutations are directly involved in UV stress-responsive pathways [48]. The UV-C-mediated activation of some transposons can be transmitted further than to one unexposed generation, which requires the involvement of Dicer-like (DCL) proteins [49]. Analysis of seed and leaf size, flowering time and transposon expression in two consecutive generations of UV-C exposed *Arabidopsis* plants revealed reduced leaf number, delayed flowering, and transcriptional reactivation of transposons in the stressed progeny. The observed changes are less prominent in the *dcl* mutants than in wild-type plants. Overall, all these studies have shown that epimutations provide an additional source of phenotypic variation that can stably persist over generations. Investigating the role of spontaneous and environmentally induced epimutations and shaping patterns of epigenetic variation is an important task to be addressed in the near future. 

### 2.2. Epigenetic Control of Plant Development 

Successful sequencing of model plants with well-annotated genomes, such as *Arabidopsis* [50], rice [51], and soybean [52], has created more opportunities for exploring the epigenome, including whole-genome methylation analysis at single-nucleotide resolution [53,54]. Comprehensive maps of DNA methylation patterns have allowed us to broaden the knowledge and understanding of the potential tissue-specific epigenetic variations and functions in plants. Widman [55] compared DNA methylation, nucleosome distributions, and transcriptional levels in the shoots and roots of *Arabidopsis* accession Columbia-0 (Col-0) and linked the observed organ-specific alterations in gene activity to particular epigenetic profiles. Despite the lack of global variations in DNA methylation levels between the studied tissues, hypermethylated genome regions tend to occur preferentially in shoots relative to roots. The same authors identified a group of genes belonging to the extensin family that have at least 10-fold higher expression and lower nucleosome density in roots relative to shoots. The biological significance of these observations has yet to be elucidated. Earlier studies of DNA methylation and demethylation patterns in four rice genotypes indicated that the relative DNA hypomethylation in roots ensure greater plasticity and adaptability to stress [56]. Although other studies have also demonstrated tissue-specific relationships between various chromatin modulators and gene expression [57,58,59,60], this topic is still a subject of debate. Specialised tissues, such as embryo, endosperm, and pollen, have shown large-scale changes in the expression of specific genes and DNA methylation patterns during *Arabidopsis* development [61,62]. In pollen and somatic cells, the methylation is maintained by comparable mechanisms; however, higher efficiency of CG methylation maintenance has been noted in pollen, which could contribute to the inheritance of methylation across generations [61]. 

Meristems that are centres for histogenesis and organogenesis are also key sites for the epigenetic control of developmental plasticity in annual, biennial, and perennial plants such as *Arabidopsis*, sugar beet, and poplar [58,59,63,64]. Meristems may mitotically or even meiotically (for shoot apical meristem) transmit epigenetic memory to new organs or to the next generation [65,66,67] or not (as in the case of vernalisation) [68]. Recent findings suggest that hormonal and redox signalling pathways interact with the epigenetic machinery to control plasticity in meristems [69], allowing the integration of epigenetic contribution in the frame of the physiological response. 

Finally, the level of overall methylation appears to be relatively stable [55,70], but it can diverge from the varying DNA methylation profiles in different plant tissues or environments [71]. Although the impact of DMRs on gene expression and phenotypic variability is often low and is still under debate [13,72,73], it could lead to natural variation of important plant traits, such as flower development [74], fruit development, ripening [75,76,77], and flavonoid metabolism [78].

Many agricultural crops are vegetatively propagated, producing clonal plants [79], which despite their clonal nature, exhibit phenotypic variability in the progeny [80,81]. Examination of regenerant lines from somatic embryos of *A. thaliana*, induced from roots or leaves, indicates that the original tissue-specific methylation and gene expression patterns are present in the sexual progeny of the regenerated plants. The epigenetic profiles and the resulting phenotypes related to the identity of the original tissue are stably transmitted during meiosis for at least four self-crossing generations [82]. Thus, the specific tissue used as explant can affect the epi-methylation and gene expression profiles of clonal plants, providing novel phenotypic variation through altered inheritance of epigenetic marks. 

Taken together, the collected information from model plants could be extrapolated to other plant species including crops, and serve as a valuable resource base for crop breeders (Table 1). 

### 2.3. Epigenetic Control of Plant Reproduction and Meiosis

Epigenetic modifications play an important role in the rate and location of crossovers, although the identification of epigenetic marks and the understanding of molecular mechanisms on pericentromeric meiotic recombination is far from being fully elucidated [83].

Germ cell development requires epigenetic reprogramming to allow a highly coordinated gene expression. Recent findings have shown that epigenetic mechanisms play an important role in key molecular and physiological processes during plant meiosis. The epigenetic modulation of plant meiosis has important implications for breeding. The transmission of epi-alleles generated in response to environmental stresses poses questions of how agronomic treatments and environmental conditions may affect the expression of key crop traits, considered in specific strategies of genetic improvement. Indeed, the understanding of the epigenetic control of plant reproduction and meiosis is of high interest from a perspective of crop genetic improvement. 

In premeiotic and meiotic anthers, 21-nt phased small-interfering RNAs (phasiRNAs) were found to be highly abundant in maize and rice [84]. *MAIZE OUTER CELL LAYER 4* (*OCL4*) encoding an HD-ZIP IV transcription factor is required for the production of small RNAs and the other 21-nt phasiRNA biogenesis in maize [85], as well as other proteins belonging to pentatricopeptide repeat (PPR) proteins, NB-LRR, and MYB families in other species [86,87]. These findings suggest that meiotic phasiRNAs play an important role in epigenetic control of meiotic chromosome condensation, with essential implications for crop genetic improvement. Mapping of genes encoding 21-nt phasiRNAs will allow the development of epi-molecular markers that could be utilised for the selection of genotypes with different rates of occurrence of meiotic events. 

Concerning crossover occurrence, it has been shown that it is positively associated with the occupancy of the histone variant H2A.Z [88]. DNA methylation and H3K9me2 lead to a repression of plant crossover hotspots [89]. Similarly, loss of DNA methylation has been shown to alter crossover distribution in a chromatin-type dependent manner in *Arabidopsis* [90]. These discoveries highlight the importance of investigating the conservative role of these epigenetic marks among crops. Future studies will identify molecular markers associated with epi-alleles, which may help to control crossover occurrence enhancing the genetic variability, and consequently, the effects of breeding selection. DNA double-strand breaks are generated by SPO11 topoisomerase-like transesterases, and SPO11-1-oligonucleotides provided a high-resolution method to profile meiotic double-strand break patterns genome-wide. SPO11-1-oligonucleotides have been mapped in the *Arabidopsis* genome and their role in regulating chromatin, DNA, and crossover frequency have been studied [91]. The identification and mapping of these short DNA sequences in crop genomes will be important to discover epigenetic markers associated with key epigenetic modulators. Another important gene involved in the epigenetic modulation of plant reproduction is *DECREASE IN DNA METHYLATION1* (*DDM1*). *DDM1* has ATPase activity that controls DNA methylation linked to crossover occurrence [92]. 

How epigenetic regulatory events can provide a source of variability by regulating chromosome dynamics during meiosis that could be usable for plant breeding is illustrated through the following examples: (i) Histone post-translational modifications: Epigenetic landscape of meiotic chromosomes highly changes during the first stage of meiosis in rice, increasing dimethylation and repressing H3K9 acetylation [93]. (ii) Small RNA and phasiRNAs: In male gametogenesis, these RNA oligonucleotides seem to be targeted towards meiocytes to allow important 3D re-organisation events at particular stages, premeioticially and in early meiosis [94]. (iii) The activity of MALE MEIOCYTE DEATH1 (MMD1) protein: MMD1 binding to methylated histones (H3K4me2) in the promoters of *CONDENSIN* genes modulates gene expression linked to chromosome condensation and meiotic progression [95]. (iv) The Argonaute protein, MEIOSIS ARRESTED AT LEPTOTENE1 (MEL1): MEL1 plays a key role in large-scale epigenetic meiotic chromosome reprogramming leading to homologous recombination and synapsis during meiosis in rice [95]. Altogether, these findings clearly demonstrated that epigenetic mechanisms are important for the control of crossing-over frequency and position along chromosomes, having a dramatic role in genetic variation within populations. This has major implications in generating genetic variability by breeding activities, as it allows better selection of parental genotypes that are utilised for artificial crossings [89]. Any future studies aiming at understanding the epigenetic mechanisms involved in the change of crossing-over events throughout the chromosomes will be of high benefit for enhancing genetic improvement. This would allow reduction of the issues of linked traits. The identification of genes and molecular mechanisms regulating recombination should be one of the hot topics for future research in plant sciences. 

Concerning gametogenesis, DNA methylation seems to be responsible for determining sexual-lineage-specific DNA methylation signatures occurring through RdDM. De novo methylation is important for modulating gene expression and splicing, which are essential for normal meiosis. RdDM activity has been shown to be involved in the *Arabidopsis* male sexual lineage controlling transcription in meiocytes. The clarification of genes and oligonucleotides involved in the modulation RdDM and their mapping in sequence genomes will be of extreme interest for developing new molecular markers associated with fertility, male sterility, and self-incompatibility. This will be very important in order to create new cultivars with desired reproductive behaviours, which will enhance the success of marker-assisted selection in breeding activity of both annual and perennial crops. The control of pollen development occurs through modifications in global DNA methylation levels from the microspore to mature stage [96]. DNA methylation is significantly different between vegetative and generative nuclei in tricellular pollen, affecting TE activity and 21nt small RNA expression [97]. The RdDM machinery plays a crucial role in female germ cell specification and imprinting, and in the establishment and maintenance of genomic imprinting [98]. Whole-genome DNA methylation events have been observed in *female-sterile* line (*fsv1*) mutants in comparison to wild-type. According to Liu and Nonomura [93], 3471 significant DMRs are observed, showing that several genes controlling ovule development and hormone responses are differentially methylated, and these epigenetic differences are linked to female gametophyte abortion [93]. In addition, *Arabidopsis* protein ARGONAUTE 9 (AGO9) has been shown to play an important role in the development of female germ cell lineages. The *ago9* mutant showed aberrantly multiplied megaspore mother cells in the developing ovule, causing unreduced megaspores [99]. 

### 2.4. Epigenetic Control of Plant Response to Stress

The epigenetic mechanisms of chromatin mark modifications and remodeling in plant stress responses (biotic and abiotic) have already been reported in multiple reviews (see recent reviews [100,101,102]).

For example, epigenetic modulation of heat-shock protein expression [103], which occurs in germ cells, may affect plant growth in adult plants [104]. Epigenetic processes are involved in heat stress responses in pollen [105]. Heat stress changes DNA methylation throughout the genome and methyltransferase expression during particular stages of pollen developmental stages [96]. Epigenetic TE silencing using RdDM in pollen can occur in response to heat stress [95]. In addition, heat stress modulates chromatin conformation through critical chromatin modifications [106] and regulates the expression of several small RNA in pollen [105]. Pollen tolerance to temperature stress is regulated by small RNAs, which are able to reprogram the male germline, repressing TEs. Some of these TEs are reactivated when the *DDM1* enzyme is mutated causing the enhanced biosynthesis of siRNAs, which work as safeguards of the epigenetic information in male gametes through the reduction of TE mobility. The elucidation of genes encoding enzymes involved in DNA methylation activity, which may affect transposon silencing, will have positive effects on pollen fertility potential, with beneficial consequences on yield in abiotic stress conditions. 

## 3. Epigenetic Advances in Crop Improvement: Exploiting Epigenetic Diversity 

### 3.1. Natural Epi-Alleles 

Naturally occurring epi-alleles have been linked to agriculturally important plant phenotypes including morphological [107,108,109,110], developmental [111,112,113], metabolic [114,115,116], and immunity traits [117]. While the origin of these epi-alleles is not always entirely clear, many of them seem to have arisen as spontaneous epimutations [34], that is, through stochastic gain or loss of DNA methylation. In crops, examples include the spontaneous hypermethylation of the *COLORLESS NON RIPENING* (*CNR*) locus in tomato, which leads to inhibition of fruit maturation [113], or the hypomethylation of the *FERTILIZATION-INDEPENDENT ENDOSPERM1* (*FIE1*) gene in rice, which is associated with reduced stature [109]. Because epi-alleles are often meiotically stable and independent of genetic variation, they present potential breeding or complementary editing targets for the agricultural sector [118,119]. Yet, examples of naturally occurring epialleles are rare, and it remains unclear how to harness them systematically for crop improvement. Epigenetic editing has the potential to introduce another source of variation that leads to phenotyping changes and an increased crop production, especially under environmental influence. Moreover, it is an alternative way to manipulate a gene by repressing or activating it for a long period of time. This gene expression modification without altering the DNA sequence itself does not lead to secondary, often uncontrolled effects, as in the case with a gene knock out during genome editing, and can be reversible and adjustable to environmental stimuli. Epigenome editing is more thoroughly discussed in the last part of this review.

### 3.2. Chemically Induced Epigenetic Diversity 

A variety of chemicals capable of modifying epigenetic information have been described. Based on their function, we have categorised them into two main groups: inhibitors of DNA methyltransferases (DNMTs) and histone deacetylase inhibitors [120]. The first group consists mainly of a cytidine analogue that specifically inhibits DNA methylation by sequestering DNMTs. Once incorporated to DNA during its replication, the analogues covalently trap the DNA methyltransferases and mediate their degradation, leading to a passive loss of DNA methylation in the cell [121,122]. The most commonly used representatives are 5-AzaC and Zebularine, but there are also more stable variants [123]. The second major group consists of histone deacetylase inhibitors, which are classified into different groups such as hydroxamic acids, amino-benzamides, cyclic peptides, and short-chain fatty acids [120]. 

Currently, DNA demethylating compounds that can de-repress the expression of the genes with hypermethylated promoters have wider applications. The most common applications are in tissue cultures, where 5-AzaC treatment has a beneficial effect on the induction of somatic embryogenesis [124,125,126], microspore embryogenesis [127,128], and shoot regeneration [129]. Another study has taken advantage of the fact that flowering is controlled by, amongst other things, DNA methylation [130]. With this in mind, the treatment of 5-AzaC has been shown to cause earlier or more frequent flowering compared to untreated control plants [131,132,133]. DNA methylation has been suggested to play a role in suppressing transposon activity. Therefore, increased activity of transposons is anticipated upon 5-AzaC or Zebularine treatment, which was confirmed by Nishimura [134], Konečná [135], and Boonjing [136]. Another group of applications focuses on the fact that the activity of the transgene in genetically transformed tissues is silenced by DNA methylation of the promoter contained in the transgenic construct. Treatment of such silenced transformants with 5-AzaC then caused reactivation of silenced transgenes [137,138]. Due to the commonly discussed role of DNA methylation in the field of priming or in transgenerational memory, treatment with 5-AzaC or Zebularine is also used for deeper understanding of DNA methylation in stress-memory-related phenomena. Verhoeven and Gurp [139] showed that Zebularine treatment affected the within-generation response to nutrient stress and indicated a role for DNA methylation in phenotypic plasticity. Rendina [140] showed that stress-induced memory alters growth of clonal offspring of white clover and that this transgenerational effect is not observed in offspring of parents that receive 5-AzaC. Similar results were obtained by Yang [141], where 5-AzaC diminished the difference between memorised and wild-type lines of *Arabidopsis*. The use of the second group of epigenetically active substances, histone deacetylase inhibitors, is currently sporadic. If they were used, it was mainly as a treatment within plant tissue cultures. Specifically, the use of Trichostatin A induced microspore embryogenesis in wheat [142] and somatic embryogenesis in *Arabidopsis* [143]. Treatment by trichostatin A and sodium butyrate also improved regeneration efficiency from mature wheat embryos [144]. It can be concluded that utilisation of epigenetically active substances is focused on topics, where it is suspected that the level of DNA methylation or acetylation of histones play a role in the respective phenomena. Specifically, this means trying to influence the various developmental stages of plants (flowering, embryogenesis, in vitro regeneration) or verifying whether DNA methylation or histone acetylation participates in a given phenomenon (stress memory molecular background). The disadvantage of such an approach is that inhibitor effects are global and not localised, affecting the whole genome and not only the locus of interest.

### 3.3. Inducing Epigenetic Diversity through Genetic Mutation

One emerging approach that aims to induce stable epi-alleles is through genetic mutations in DNA methylation pathways [145]. In the model plant *A. thaliana*, many studies have shown that DNA methylation mutants exhibit altered phenotypes, which are due to trans-induced hyper- or hypo-methylation of specific gene loci [146,147,148,149,150]. While many of these induced traits appear to be deleterious, with varying degrees of penetrance [148,149,151], several examples do exist where induced epi-allelic variation leads to advantageous phenotypes [152,153,154]. For instance, in *Arabidopsis*, mutants in RdDM displayed enhanced resistance against biotrophic pathogens, such as the downy mildew pathogen *Hyaloperonospora arabidopsidis* [152]. In addition, reduced DNA methylation levels in *Arabidopsis* DNA methylation mutants flowered earlier without the requirement for vernalisation [154], an agronomic trait that is usually preferable over late flowering. In the model tree poplar (*Populus* spp.), hypomethylation through a mutation in *DDM1* increased tree tolerance in response to water deficit, showing how *DDM1* context-dependent DNA methylation plays a crucial role in tree phenotypic plasticity under drought stress conditions [155]. The authors suggest major roles for DNA methylation in the shoot apical meristem in regulating genes involved in hormone-related stress responses, and the maintenance of genome integrity through repression of TEs.

Importantly, these phenotypes can sometimes persist for many generations, even when the inducing mutations are segregated away [156,157,158], indicating that they are acquired through epigenetic variation. This latter observation has been exploited systematically in the construction of so-called epigenetic recombinant inbred lines (epiRILs) [156,157]. This experimental system is derived by crossing a DNA methylation mutant to wild type. The F1 progeny of this cross are either backcrossed to wild type or intercrossed, and individual homozygous wild type for the inducing mutation are repeatedly self-pollinated to derive recombinant inbred lines. The final plant material thus consists of isogenic, epi-homozygous individuals, whose methylomes are a mosaic of the two original parents. These novel epi-genotypes can be systematically screened for favourable traits. Two epiRIL panels have been created in *A. thaliana* using *DDM1* [156] or *MET1* gene [157] as inducer mutations. 

Extensive phenotypic surveys of the *ddm1*-derived epiRILs have revealed large heritable variation for key agricultural traits, such as plant height, root length, flowering time, rosette size [156,159,160], pathogen resistance [153], and phenotypic plasticity under salt stress [161]. Heritability estimates are in a range similar to what is typically seen in crop breeding [159,162]. By design, the epiRILs are also amenable to epigenetic quantitative trait locus (QTLepi) mapping approaches, where DNA methylation markers are used in genome-wide linkage scans [159]. Numerous epigenetic QTL have been identified. In some cases, the mutant epigenotypes at the QTL locus are associated with improved phenotypic performance relative to wild type, indicating that the underlying epialleles could be exploited in breeding programs [153,159,161,163]. More recently, epiRILs have also been utilised to generate large panels of so-called epiHybrids by crossing selected lines to wild type [164,165]. The epiHybrids display substantial best-parent heterosis for important traits like early vegetative growth [165], early flowering time, increased leaf area, plant height, and main stem branching, several of which could be linked to differential methylated regions in the parental genomes [164].

There have been efforts in the academic and private sectors to implement similar epigenetic perturbation strategies in crops. Such approaches could complement more mainstream breeding tools focused on genetic variation. However, the choice of inducer mutation is more challenging here, since loss of *MET1* or *DDM1* tend to be lethal or cause severely reduced fertility in complex crop genomes [166,167,168]. A promising alternative candidate is the *MSH1* system. The *MSH1* mitochondrial and plastid-targeting protein is involved in genome stability and influences plant growth patterns [169,170]. By crossing *MSH1* to wild type, *MSH1* is suppressed by RNA interference, resulting in enhanced plant growth and heritable epigenetic changes in *Arabidopsis* [171], as well as in tomato [19]. Notably, the methylome reprogramming in *Arabidopsis* is accompanied by changes in siRNA expression and heritable non-genetic memory [141]. In addition to crosses, the *MSH1* system has also been employed in grafting experiments to introduce rootstock epigenetic variation, where the progeny also displays increased seed yield and growth vigour [20,171]. Most importantly, the *MSH1* system seems to be reproducible in tomato field size experiments, making it a promising system for agricultural applications.

### 3.4. Environmentally Induced Epigenetic Diversity

Plants are immobile and exploit various tools to survive and reproduce under suboptimal or harsh surroundings. In the case of environmental stimuli, they undergo physiological response and phenotypic changes, which, according to the length of exposure, are divided into developmental plasticity (short term stimuli), memory and priming (mid-term stimuli), and local adaptation (long term exposure) [172]. There are many examples shown above that plants are better prepared for repetitive exposure to stress, either in a given individual, a derived clone, or even transgenerationally [173]. It is therefore possible to expect that similar mechanisms of plant adaptation also occur in situations where plants are exposed to natural stress conditions. Despite the relatively well-described and known memorising effect, the unique properties of plants that are naturally adapted to their respective environment is not yet very widespread in practice. One example is the fact that plants in different environments produce different ranges of secondary metabolites [174], which may theoretically be useful in the field of medical plants or spices [175]. From the evolutionary point of view, the issue of environmentally induced changes indirectly includes the strategy of vernalisation as a possible way for plants to adapt to extreme winter frosts [176] or for the epigenetic background of invasive species to successfully establish in different environments [177,178].

#### 3.4.1. Epigenetic Stress Memory and Priming 

**Figure 2:**
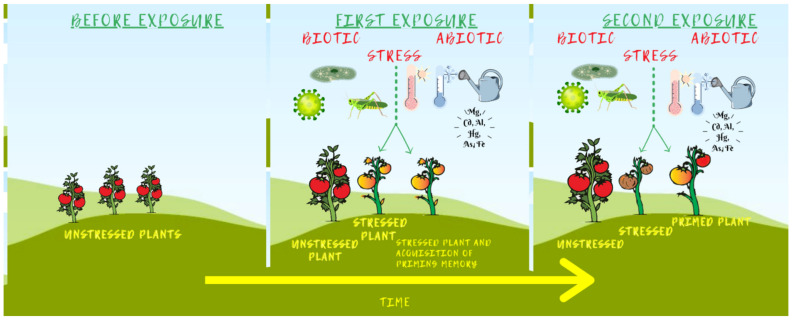
Intragenerational memory and priming in plants. Unstressed plants (tomato as an example of crop) are depicted on the left panel. One of them is shown growing over time in the other panels on the left with red healthy fruits. Stressed plants with the first exposure to biotic and/or biotic stimuli are shown on the middle panel with orange fruits and decreased size. The plant on the right developed an epigenetic response to stress, allowing the establishment of a priming memory. Stress is only temporary and is followed by a period of recovery (not shown). A second exposure to stress is shown on the right panel, with the primed plant (on the right) getting new red fruits and restart of growth compared to the prime plants (in the middle) exhibiting severe symptoms (brown fruits).

Although we tend to think that plants need to adapt to unpredictable environments, many of the environmental cues and stresses that a plant experiences throughout its life are recurrent. This is the case for daily changes (e.g., decrease of temperature at night) and seasonal changes (e.g., increase of temperature in spring). It is frequent that plants and their offspring must cope with repeated similar stresses—whether they occur throughout the growing season or over successive plant generations. These climatic patterns can lead to the so-called stress priming, that is, a first encounter with the stress can trigger the establishment of a molecular memory that primes or acclimates the plant and/or its offspring, which will be better prepared to respond in the eventual case of a second stress (Figure 2). This priming effect induced by stresses, also known as acclimation when the trigger is of abiotic nature, (i) is conserved, (ii) can be triggered by stresses of different nature (i.e., biotic or abiotic), and (iii) can occur intra-generationally (the molecular memory can be transmitted to new cells and organs) and inter/transgenerationally (the memory is passed to the offspring). In addition, priming has been shown to be effective at different stages of the plant life cycle, from seed (i.e., seed priming) to seedlings and to subsequent adult stages. However, while much is known about the physiological consequences of priming in the adaptability of the plant and hence its applications—products that boost plant vigour through priming are available from different agricultural companies—much less is known about the molecular mechanisms that establish, regulate, and even erase this molecular memory [179,180,181,182].

Although metabolic and physiological pathways have been shown to play a role in priming, it has been proposed that a long-lasting process able to be transmitted through cell division, such as epigenetics, could be key in establishing the primed memory [183,184,185,186]. More recently, the molecular evidence supporting this hypothesis in diverse plant species has brought a novel focus on this field [179,180,187].

When plants are exposed to stress, a transcriptional response is triggered in response to the stress. Among the stress-responsive genes, some of them will be involved in establishing a memory of the stress and are, therefore, known as stress memory genes. Isabel Bäurle, who has extensively contributed to our understanding of heat stress priming in *Arabidopsis*, has proposed that memory genes can be classified considering their transcriptional response to the stress. In type I memory genes, the epigenetic changes induced by the stress will induce the maintenance of the new transcriptional state posed by the stress during a certain period of time, while in type II memory genes, epigenetic marks that are established on the chromatin of the gene during this first encounter, will induce a modified transcriptional response to a second stress, usually stronger and faster than the first one [179,180]. For intra-generational memory, the lapse of time between the repeated stresses seems to be limited to a few days or, occasionally, several weeks [188,189]. For trans-generational memory the repetition of the stress in successive generations is key for keeping the transcriptional state associated with the primed response [46,190,191] and a stress recovery phase of the mother plants may be crucial [191]. Although priming has been well-studied in annual plants, such as *Arabidopsis* [179] or the model crops maize and rice [172,192,193], the molecular memory induced by stress may indeed be particularly relevant for perennial species, among them economically important crops such as poplar [65,66,194], and clonally propagated plants, for instance grapevine [195]. In addition, maintaining the primed state required to surpass the energetic costs, otherwise resetting the memory, may be more advantageous for the plant [181,187,191].

Both DNA methylation and PTMs have been linked to the primed transcriptional changes in memory genes, although DNA methylation may be the key epigenetic mark for mediating trans-generational memory, as it may be more stable to the epigenetic reprogramming that occurs during gamete formation [179,181]. Among the histone modifications related to priming, changes in the repressive mark H3K27me3 and, particularly, in the active mark H3K4me3 have been shown to occur on memory genes in response to different stresses. RNA biology, such as small RNAs, RNA turnover, and RNA-mediated epigenetic changes, can also mediate the memory to past stresses [179,189,196]. Chromatin remodelers able to alter DNA–histone interactions add another layer of regulation to the primed transcriptional response [179]. Finally, factors involved in the deposition of nucleosomes and the inheritance of epigenetic states into new DNA molecules have been involved in perpetuating the stress memory [179,187].

It is tempting to speculate that a better understanding of the epigenetic mechanisms that govern the establishment of a priming memory may contribute to developing novel molecular technologies for crop stress adaptation improvement without affecting plant fitness. However, this will still require a deeper knowledge of intra and trans-generational memory events in response to multifactorial on-field conditions and further development of transient or stable transformation methods able to reprogramme the epigenome of stress memory [197,198]. The importance of including primed responses to develop predictive models of crop fitness and adaptation to environmental changes is further discussed in Section 4.

#### 3.4.2. Clonal Propagation and Uses

An additional area where environmentally induced changes are becoming important is the clonal propagation of plant species such as perennial woody plants, especially if they are grown in a broad range of environmental conditions. As clonal plants, they provide a great opportunity to study the effects of epigenetics without concern for genetic variation. Different climatic conditions establish locally unique epigenetic marks that can be theoretically used for the study of epigenetic mechanisms underlying plant–environment interactions or phenotypic plasticity. Guarino [199] studied changes that occur in the epigenome of ramets of the same poplar clone living in different natural environments of Sardinia. Analyses clearly demonstrated that ramets of the same clone were differentially methylated in relation to their geographical origin, that is, in response to environmental stimuli. A different approach was used by Schönberger [200], who collected clonal *Populus trichocarpa* (cv. Muhle Larson) cuttings from two sites in Germany that differ in phosphorus availability. Clones were subsequently transferred into a common, fully nutrient-supplied environment. Despite identical underlying genetics, stem cuttings derived from sites with lower phosphorus availability showed impaired establishment. Simultaneously, genome-wide DNA methylation differences between variants were identified. Vanden Broeck [201] also investigated the establishment of a Lombardy poplar (*Populus nigra* cv. Italica Duroi), a cultivated tree representing a single genotype worldwide distributed since the eighteenth century, collected from 37 locations in Europe and Asia with different environments. The collected cuttings were transferred to common conditions where bud set and CG methyl polymorphisms were studied on one-year-old offspring. Correlations among epigenotype, bud phenology, and the climate at the home site of the donor trees were observed. Pereira [202] compared patterns of DNA methylation of biological replicates of leaf and xylem tissue samples from four commercially planted elite Eucalyptus grandis × Eucalyptus urophylla clones and one Eucalyptus urophylla in two contrasting sites in Brazil by using high-throughput MS-DArT-seq (Methyl Sensitive DArT-seq sequencing). Association of methylation patterns proved by using MS-DArT-seq was registered for the respective environments and growth traits. All of the above examples confirm that the DNA methylation landscape usually differs in clonal materials grown in different environmental conditions. The question therefore arises as to whether, on the contrary, different DNA methylation profiles could be useful in determining the origin of a given plant material. If this hypothesis was confirmed, it would bring a relatively valuable tool to the field of authentication of the origin of plant products. The first article on this field presented by Xie [203], who studied genetic and epigenetic diversity across 22 vineyards planted with the cultivar Shiraz in six wine sub-regions of South Australia. It was observed that DNA methylation differences between vineyards are influenced by the geographic location where the North–South axis of the topology significantly contributed to the variability. The pruning system used in individual vineyards also contributed to the observed variability but to a lesser extent. For the first time, the idea was presented here that it would be possible to use that epigenetic variability as a tool to track terroir of grapevines. Baránková [204] indirectly proved this hypothesis when they compared DNA methylation patterns among clones of Merlot and Pinot Noir varieties planted in completely different climatic conditions in the Czech Republic (Central Europe) and Armenia (Southern Caucasus). The DNA methylation landscapes (79.9% and 70.7% for Merlot and Pinot Noir plants, respectively) were primarily affected by the different geographical locations of the Czech and Armenian vineyards. On the other hand, DNA methylation diversity within individual vineyards of Merlot and Pinot Noir cultivars represent only 16% and 14% of the overall diversity and suggest uniqueness of DNA methylation landscapes for individual vineyards. The phenotypic traits and epigenomes of three clones of cv. Malbec cultivated in two contrasting vineyards in Argentina were compared by Varela [205]. They concluded that DNA methylation has an important role in the phenotypic plasticity and that epigenetic modulation was more clone-dependent than location-dependent. Thus, on the basis of published literature, it is clear that additional follow-up studies comparing more factors (for example different tissues, growing seasons, geographical locations) need to be performed to verify if DNA methylation can be utilised as a marker for terroir authentication.

### 3.5. Hybridisation and Epigenetic as a Predictive Marker of Hybrid Performance 

Heterosis, or hybrid vigour, is the superior phenotypic performance of F1 hybrids compared to the two inbred parents [206]. The phenomenon is extensively exploited to increase crop production worldwide [207]. Breeding programs aim to optimise heterosis by carefully selecting parental genotypes for crossing. However, genotypic information is often insufficient to accurately predict heterosis, and the molecular basis of this phenomena remains poorly understood [207]. Accumulating evidence points to a strong epigenetic contribution to heterosis. Molecular profiling of hybrids shows that their epigenomes are substantially remodelled with respect to their parental lines, leading to epigenetic states that diverge both positively and negatively from the expected mid-parent values (MPV) [208,209]. Extensive remodelling has been observed at the level of DNA methylation in *Arabidopsis* [164,208,209,210,211], rice [212,213], pigeon pea [214], broccoli [215], and in rapeseed [216], and occurs either at regions where parents are differentially methylated (DMRs) [164,208,209,217] or in regions where the parents are similarly methylated (SMRs) [213,215,218]. Similar observations have been made at the level of small RNA (sRNAs) abundance [196,208,210,212,214,216,218,219,220,221,222,223,224,225] and at various histone modifications [212,226]. In many cases, these epigenetic changes lead to downstream non-additive gene expression levels, which have been linked to heterotic phenotypes [118,208,209,212,214,216,219,227,228], often associated with salicylic acid (SA) metabolism and response [218,229]. For example, differences in the SA endogenous level in F1 heterotic hybrids have been correlated with enhanced leaf growth in *Arabidopsis* [229], and Zhang [218] not only revealed *DDM1* as an epigenetic link between growth vigour and SA metabolism but also suggested that parental SA differences can predict heterosis level in the F1.

While it is possible that the remodelling of hybrid epigenomes is “just” the molecular consequence of classical genetic complementation effects, recent evidence shows that it also occurs when the parental genomes are isogenic or nearly isogenic [164,165,208,210,211,230]. This suggests that non-additive epigenetic changes observed in hybrids may originate from interactions between divergent parental epigenomes. Given the close association between DNA methylation, PTMs, small RNAs, and gene expression, parental epigenetic differences may therefore be used as indicators of hybrid performance, independently of genetic differences. Lauss [164] demonstrated that this is indeed the case. Using a panel of *A. thaliana* epigenetic Hybrids (epiHybrids) obtained from crosses of isogenic but epigenetically divergent parents, they showed that heterosis for flowering time, height, and leaf area could be associated with specific DMR between the parents. Similarly, in canola, hybrids obtained from isogenic parents which had undergone prior divergent selection for high energy-use efficiency showed increased yield. The artificial selection of the agronomical characteristics and the distinct DNA methylation parental patterns were heritable in the hybrids and allowed the targeted increase of yield [231].

These results show there is predictive information in epigenetic markers beyond DNA sequence variation. Epigenetic measurements may therefore be used as complementary biomarkers, even in crop systems where the parental lines are highly polymorphic. To illustrate this, Seifert [220] quantified differences in sRNA expression profiles among different heterotic groups in maize. The parental divergences in sRNA were strong indicators for grain yield in the hybrids [220,232]. The magnitude of these predictions exceeded those obtained with polymorphism data [220,232].

Beyond heterosis, epigenetic markers have been used to associate epigenetic with trait variation in experimental and natural populations. Classic quantitative genetics methods used to estimate the genetic component of the phenotype could also be used to assess natural epigenetic variations. DNA methylation represents a trait allowing the estimation of narrow-sense heritability or genetic differentiation index describing the genetic control of epigenetic variation [66,233]. Epigenetic polymorphisms (SMPs or DMRs) could be used as epigenetic markers in genome-scan-based approaches to estimate the role of epigenetics in population structure and phenotypic variation (epiFST, epiQTL). In oilseed crop using stable and heritable methylation-sensitive amplification polymorphisms (MSAP) and retrotransposon (RT) epigenetic markers, Long [234] identified QTLs for seven agronomic traits. In a similar MSAP-based approach in sorghum, a methylation linkage map was constructed that could help associate methylome hotspots to elite genes responsible for traits [235]. A common question is whether epigenetic variation is a cause of differentially expressed genes or the consequence of it. Recent evidence from maize supports the former, as DMRs that are not tagged by genetic variation are more prevalent and strongly associated with the expression of genes identified in phenotyping variation [78]. In addition to this, many metabolic traits showed notable correlations with DMRs and not with single nucleotide polymorphisms (SNPs), thus highlighting DNA methylation variation as a powerful phenotypic predictor independently of genetic polymorphism data [78]. Epigenome-wide association-mapping approaches have also been employed in clonally propagated oil palm. This approach uncovered a recurrent epimutation, which could be shown to be responsible for parthenocarpy and loss of oil yield [108]. Local hypomethylation near the Karma transposon could be shown to predict deleterious phenotype and is being further developed as a biomarker for juvenile screening of trees. 

Beyond statistical predictions, knowledge of epigenotype–phenotype associations may provide deeper insights into the molecular regulation of agriculturally important plant traits and has the potential to contribute to breeding with useful markers. Even though there are already several studies in model plants that were able to quantify the epigenotype–phenotype interactions by distinguishing between the DNA sequence and epigenome variation, a defined epigenetic-oriented breeding strategy is yet to come. The creation of such systems in crops would allow not only the targeted use of the epigenome diversity to detect agronomically important epialleles, but also has the power to predict a plant’s performance, decrease field trials costs, and result in an increased crop production.

## 4. Gaps in Knowledge and Future Challenges

### 4.1. Improving Strategies for Studying the Role of Epigenetics in Crops under Changing Environment

Most studies on crops have focused on developmental and/or environmental interactions with epigenetic marks (particularly DNA methylation), mainly through descriptive or correlative approaches between agronomic traits and epi/genomics data (Table 1). Recently, Alonso [236] proposed suitable approaches to improve plant functional phenotypic traits for biotic stress. The authors proposed (i) “concurrent analysis of epigenetic variation and phenotypic trait variation, including plant fitness between individuals exposed to contrasted biotic interactions” and (ii) “analysis of specific loci and physiological pathways to clarify the epigenetic contribution to the stabilisation of environmentally induced phenotypes (priming) or across generations’’ to gain insights into functional relationships. In addition to these recommendations, Amaral [194] proposed a complementary methodological plan for tree breeding that can be easily extended to crops, including (i) use of both forward and reverse (epi)genetic approaches and development of population epigenomics; (ii) assessment of the effects of multiple, potentially interacting, stressful conditions (intensity, duration, frequency, interaction); (iii) favour of field experimental designs; (iv) use of kinetics approaches by sampling biological material along a developmental gradient for a better understanding of the molecular chain acting from short to long term during development and environment interactions; (v) taking into account crop features (species, genotype or variety, physiological and chronological ages, organs, tissues), but also the geographic origin, clone or seed history, clonal propagation vs. sexual reproduction, and all features of breeding, management of genetic resources and culture; and (vi) development of trans-omics approach to overcome the lack of comprehensive understanding and the information gap regarding interaction across multiple -omic layers to move from correlative to causal inference and predictions. 

### 4.2. Modelling Epigenetically Regulated Complex Traits in Crops

#### 4.2.1. The Need to Link Epigenetic Marks to Phenotypes into Modelling Frames 

Although epigenetic marks have been the matter of prediction in many recent studies, most modelling approaches concern human health studies, for example computational modelling of histone modification, DNA methylation, and their interdependency in environmentally induced cancer initiation or neurodegenerative and autoimmune diseases [230,231,232]; and age prediction for which the degree of cytosine methylation in specific genes could help estimating the age of a person, thus predicting the risk of age-related disorders [237] or complex human traits (such as body mass index, lifestyle factors) using genome-wide DNA methylation profiling [238]. This approach has been much less undertaken in plant science, and so far, most efforts were devoted to the development of statistical and mathematical models to link DNA methylation profiles at the promoter region of specific genes to a phenotype (e.g., prediction of plant height in *Arabidopsis* in [239]) or to identify parental epigenetic markers to predict characteristics in hybrids (commented on in Section 3.5, [240]).

Epigenetic regulating marks were shown to be involved in a wide range of plant agronomic traits, irrespective of environmental cues, such as vernalisation [241,242], plant height [156], seed development [243], nodule formation in legumes [244], and lycopene production in fleshy fruits [75]. Therefore, improving crop features requires being able to predict these agronomic traits and/or developmental behaviours by taking into account the underlying epigenetic functions for prediction purposes. For example, bolting tolerance in sugar beet was shown to be a genotype-dependent control of DNA methylation and expression of an integrative gene network [63]. To our knowledge, however, the incorporation of epigenetic regulatory mechanisms into modelling frames or even into process-based crop models is almost non-existent. The following two examples illustrate that modelling approaches have been published but are still scarce. One of the first modelling studies deals with the well-known process of vernalisation that allowed the quantitative prediction of epigenetic silencing of the gene encoding the floral repressor FLOWERING LOCUS C (FLC) in relation to the duration of cold exposure [241,245]. Another pioneering attempt concerns lycopene production in tomato [246]. Based on a detailed description of the ripening processes at the molecular level, where the silencing of *DEMETER-LIKE DNA DEMETHYLASE (DML2)* was shown to impair DNA methylation levels and thus hamper carotenoid accumulation, a predictive model of lycopene production was developed. It relies on the interplay between the key ripening-related transcriptional factor NON RIPENING (NOR), the PHYTOENE SYNTHASE 1 *(PSY1),* and the level of DNA methylation at their respective promoters [75]. This example highlights the importance of deciphering the cascade of complex epigenetic regulations, which is the prerequisite to building process-based models (i.e., models that rely on specific mechanistic steps and their interactions) dedicated to the prediction of agronomic traits irrespective of adaptive responses. Associated with this challenge is the required transfer of epigenetic knowledge acquired from model plants towards crops.

#### 4.2.2. Modelling Epigenetic Regulation Induced by Environmental Stress 

For the last decades, predictive modelling has become a central tool to help agronomists and breeders orienting towards key adaptive crop traits to climate change. Indeed, the use of crop models has been extended to climate-change-driven predictions [247,248,249,250,251,252,253]. Crop models which were originally designed and implemented to take into account environmental constraints such as light and temperature but also fertilisation management (e.g., APSIM [254], STICS [255], SUCROS [256], DSSAT [257], CROPGRO [258]) need to be updated and redesigned to carefully consider new features of climate change (i.e., higher frequency of extreme events such as heat waves, frost, drought, flooding or salinity). Indeed, the repetition of extreme events highlights the question of stress memory (i.e., the ability to store and further retrieve the information induced by a first stress exposure supported by, among others, epigenetic mechanisms, which can be beneficial to plant performance and adaptation under recurrent stresses [179,189,196]). 

**Figure 3:**
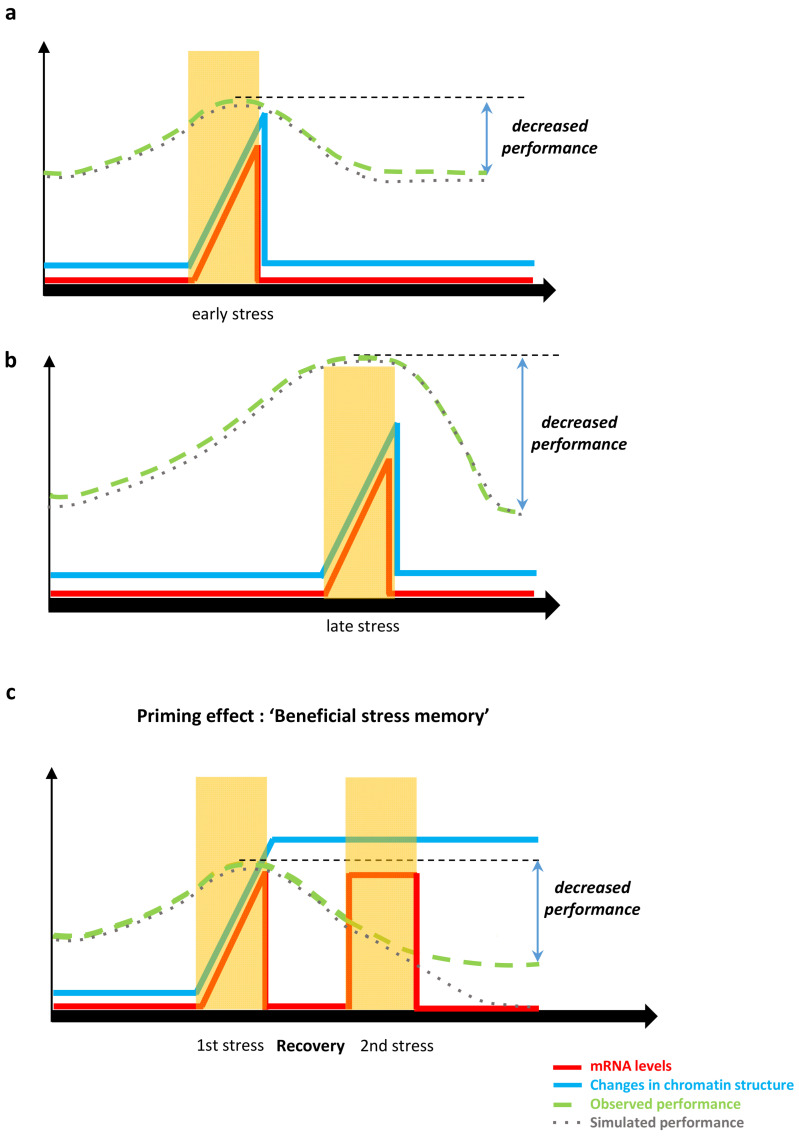
Illustration of the above-raised modelling questions when simulating stress response (measured through plant performance) in a two-stressing-event sequence when a priming effect from the first stressing event is observed. Graphs (**a**) (green dotted line) and (**b**) (green dotted line) display negative effect of a single stressing event (early (**a**) and late (**b**)) on plant performance along with associated molecular events (levels of RNA transcripts and chromatin structure changes). Graph (**c**) (green dotted line) shows how priming alleviates the negative effect of the first stress exposure on the plant performance through the maintenance of chromatin changes, allowing faster (sometimes more efficient) gene transcription and hence plant adaptive response. Simulated performance under the two-stressing-event sequence (graph (**c**), grey dotted line) might result in the addition of the two decreases that followed each single event if the model does not take into account the stress memory effect. Adapted from [270].

Therefore, the climatic context prompts one to take into account the stress factors that trigger the epigenetic changes which contribute to the beneficial effects of stress memory on crop performance throughout the growing season or over successive generations. Although breeders have already seized the challenge [118,259,260,261], crop modellers are urged to do it in order to avoid growing modelling biases in the prediction of crop yield and to improve quality of harvested products in the near future [251,262,263,264]. Crop models are usually calibrated and evaluated with datasets that do not focus on successive stresses, but on long-lasting stresses (e.g., mineral limitation, increased temperature over one specific phenological stage). Therefore, the emerging question concerns the extent of biases in the responses of crops that are challenged to a long-term stressing period vs. to repeated stressing events as observed when plants acquire stress memory. Indeed, when two stressing events occur, the modelled plant response will result in two levels of decreased performance which do not represent the plant behaviour when the first stressing event triggers beneficial stress memory (Figure 3). Another example of model disruption deals with the characteristics of seeds from stressed mother plants. Studies on several crops indicated that germination kinetics were modified when seeds came from heat- or drought-stressed mother plants [265,266], thus leading to a lower value of the base temperature for germination and to faster seed germination kinetics under higher temperature (Brunel-Muguet, unpublished). This is in line with prior observations which indicated that landraces (or ‘peasant seeds’) are more resilient to extreme climate events than varieties produced under semi-controlled or homogeneous and stable conditions [267]. For crop models where the base temperature for germination is a key plant parameter (e.g., SIMPLE) [268,269], unchanged value will result in prediction biases. This example highlights the evidence for trans-generational memory of heat stress that can modify plant parameter values in a crop model.

In conclusion, crop improvement under ongoing and future climate change relies on our ability to predict plant behaviour (i.e., the magnitude and direction of stress responses, the mechanisms of which are supported by epigenetic regulations). Therefore, process-based models could be the cornerstone of the prediction of epigenetically controlled adaptive traits, and their implementation and redesign open up a promising field of investigation for crop improvement under climate change.

### 4.3. Biotechnologies and Epi/Genome Editing

A key challenge in agriculture of the 21st century is to speed up crop breeding and the development of new varieties, with higher yield, superior agronomic traits, and more resilience to climate changes in a context of increasing demand for global food security. In forestry, improvement programs search for trees that are better adapted to biotic and abiotic stress conditions as temperatures increase. In vitro plant regeneration systems are essential in modern breeding techniques, since they permit one to clonally propagate elite genotypes (through somatic embryogenesis), to produce double-haploids with new genetic variability (through microspore embryogenesis), and to perform gene editing or transformation (which require efficient plant regeneration methods). 

#### 4.3.1. Targeting Epigenetics for In Vitro Regeneration to Improve and Accelerate Crop Breeding 

Biotechnology methods for in vitro plant regeneration and breeding are based on the exploitation of the potential for cell reprogramming, a process where epigenetic regulation plays a key role, which is not yet completely understood [271,272]. Conversely, in vitro culture is known to induce somaclonal variations with genetic and epigenetic instabilities. Recently, Ghosh [273] reviewed these aspects with respect to DNA methylation and crop improvement. Through in vitro embryogenesis, somatic cells can be reprogrammed, giving rise to embryos and plants, without the fusion of gametes [274]. If the microspore (haploid cell, precursor of pollen grain) is reprogrammed towards an embryogenic pathway [275], haploid embryos are developed and, after spontaneous or chemically induced diploidisation, doubled-haploid (DH) plants are produced, permitting one to accelerate the breeding process. With DH technology, completely homozygous plants can be established in only one generation, while in a conventional breeding programme the development of homozygous lines normally involves several generations of selfing and selection [276]. Somatic embryogenesis, induced from vegetative tissues, has been demonstrated to be very useful for large-scale clonal propagation of selected genotypes, with improved traits, in species with long reproductive cycles, like many fruit and forest trees [271], as well as for the production of genetically modified and, more recently, gene-edited plants.

Somatic embryogenesis also constitutes a unique system for studying the epigenetic memory and its influence on adult plant fitness and adaptation. One of the best examples has been documented in *Picea abies,* where epigenetic memory marks can be established in response to the (high) temperature conditions imposed during somatic embryogenesis, being the epitype fixed during embryo formation and mitotically propagated to the adult tree [277]. Epigenetic memory formed in the somatic embryos permanently affects several important adaptive traits of the tree, improving its adaptation to higher temperatures. Interestingly, formation of the temperature-induced epigenetic memory in somatic embryos of Norway spruce is associated with defined transcriptional changes that include differential expression of specific epigenetic regulators, writers and erasers of epigenetic marks [278]. Transcriptomic analyses enabled the identification of a number of epigenetic regulators involved in the process, and supported the key role of DNA and histone methylation and sRNAs to establish an epigenetic memory in *P. abies* [278].

Despite the important role of in vitro plant regeneration systems in breeding techniques, plant cell reprogramming and in vitro embryogenesis systems still show very low efficiency in many crop and forest species, as technical innovations in this field are hampered by our incomplete understanding of the molecular mechanisms underlying these processes. Increasing evidence indicates that together with hormones, epigenetic mechanisms play an important role in somatic embryogenesis induction and progression [275,279,280]. This data opens the door for targeting epigenetics as a potential biotech strategy to improve and accelerate crop plant regeneration and breeding [66,194]. For these studies, methodologies to monitor changes in global DNA methylation levels and nuclear patterns have been very useful [281,282]. Recent reports have shown that cell reprogramming and embryogenesis initiation require widespread DNA hypomethylation [96,283], histone H3K9 and H3K27 demethylation [179,284], and histones H3 and H4 acetylation [285,286]. Moreover, chromatin-modifying enzymes, like DNA METHYLTRANSFERASES, HISTONE LYSINE METHYLTRANSFERASES and DEMETHYLASES, and HISTONE ACETYLTRANSFERASES and DEACETYLASES have been proposed as modulators of plant cell reprogramming that act by changing the distribution of some epigenetic marks and promoting an open chromatin state [287]. It has been suggested that these changes in epigenetic repressor marks would lead to the expression of specific transcription factors which would activate auxin signalling and regulatory feedback loops, which would eventually trigger embryogenesis [288,289,290].

Recent advances in chemically controlled mammalian cell reprogramming have shown the high potential of application of synthetic small molecules, including epigenetic inhibitors, to control cellular reprogramming, proliferation, and differentiation, as well as cancer cell reprogramming [291,292]. In plants, epigenetics chemical targeting with inhibitors of DNA methylation (5-AzaC, Zebularine), H3K9 methylation (BIX-01294), or histone deacetylases (Trichostatin A, Suberoylanilide hydroxamic acid, SAHA) has been demonstrated to improve in vitro plant cell reprogramming and regeneration, to produce DHs, and to propagate selected clonal plants in a number of crop and forest species, such as rapeseed, barley, wheat, and cork oak [128,142,284,286,293,294].

Another plant biotechnological application where our knowledge in epigenetics can be exploited is the production of secondary metabolites for industrial use. Recently, an integrative analysis has unravelled the role of DNA methylation in the developmental and environmental control of specialised metabolisms (alkaloids) in a medicinal plant (*Catharanthus roseus*) [71]. One possibility will be to exploit the potential coordination between epigenetics and hormonal control [69] to improve the production of secondary metabolites for pharmaceutical applications using plants.

Some of the potential future challenges are to identify new elements of the regulatory epigenetic pathways controlling plant cell reprogramming and regeneration, together with the design and synthesis of novel small compounds and chemical libraries for epigenetic targets, which will pave the way for new biotechnological strategies by using small cell-permeable synthetic molecules to enhance in vitro plant regeneration yield. Furthermore, research on the development of high-throughput screening and phenotyping assays to test epigenetic modulators from newly created chemical libraries will be necessary to efficiently exploit epigenetic targeting approaches to improve the efficiency of in vitro embryogenesis systems for accelerating crop breeding to adapt to climate change, even in recalcitrant species. 

#### 4.3.2. Epigenetic Editing

As discussed here and in other reviews, many epigenetic factors play a regulatory role in multiple developmental and physiological processes by directly affecting chromatin organisation and gene transcription. Therefore, modification of epigenetic factors harbours the potential for crop improvement, namely epi-breeding. Similarly to genetic editing for precise genetic modifications, epigenetic editing refers to a targeted change of a specific epigenetic mark (i.e., methylated cytosine or histone tail methylation, acetylation, etc.) at a predefined genomic site, mostly in order to modulate transcription. Unlike genetic editing that is already commercially implemented in tomato [295], epigenetic editing is still far from being applied in crop species, however several publications demonstrate its feasibility in *Arabidopsis*. Using programmable DNA-binding proteins fused to different components of the RdDM machinery, DNA methylation was induced specifically at the *FWA* target locus [296]. *FWA* is a flowering inhibitor in *Arabidopsis*, normally silenced due to DNA methylation of tandem repeats in its promoter, leading to early flowering. In the fwa epi-allele, the lack of methylation on its promoter results in delayed flowering and extended vegetative phase [148]. This system was utilised to study the effect of DNA methylation on gene expression and to develop new tools for DNA methylation editing [297]. Johnson [298] fused *SUVH2* to a zinc finger (ZF) protein designed to target the *FWA* promoter and showed that this fusion is capable of recruiting PolV to the target site and inducing DNA methylation. ZF fusions were shown to induce methylation at the FWA target also when joined with other RdDM components, such as SHH1, NRPD1, RDR2, DMS3, and RDM [296]. In addition, a fusion between a ZF and the catalytic domain of the human demethylase TEN-ELEVEN TRANSLOCATION1 (TET1) caused highly efficient demethylation of the *FWA* promoter, transcriptional up-regulation, and a heritable late-flowering phenotype. Other ZF–TET1 fusions designed to target methylated regions of the *CACTA1* transposon were used to induce targeted demethylation and changes in expression [299]. The recently developed CRISPR/dCas9 system enabled the targeting of the TET1 catalytic domain to the same loci, inducing targeted demethylation and activating gene expression [299], as well as inducing alternation between two epi-allelic states at a specific locus [300]. The CRISPR/dCas9 tool was further adapted to induce targeted methylation using the catalytic domain of the Nicotiana tabacum DRM methyltransferase (NtDRMcd), which was known to be well-folded and well-expressed [198,301]. This system enabled an efficient and specific induction of DNA methylation at the *FWA* and *SUPERMAN* loci, affecting gene transcription [198].

In order to harness these epigenetic editing tools for crop improvement, we need to identify specific targets and define the specific mark(s) linked with traits of interest. These targets should exhibit a specific, stable epigenetic feature at a well-defined genomic locus. This is a challenging task, mainly due to the dynamic nature of epigenetic features. Few genetic elements that are related to an agricultural trait and are controlled by DNA methylation were identified in different crops. A naturally occurring epi-allele that accumulates high levels of vitamin E in tomato was associated with differential methylation of a *SINE* retrotransposon located in the promoter region of the *VTE3* gene [115]. In maize, the ZmMI1 element is transcribed only under cold stress accompanied by changes in DNA methylation patterns [302], and in allo-tetraploid cotton, the *COL2* epi-allele affects flowering time, which is linked with changes in DNA methylation [303]. Such genes are possible candidates for DNA methylation editing for crop enhancement (Table 1). In addition, accumulating epigenomic data in various crops will facilitate the identification of additional candidate targets. For example, genome-wide changes in DNA methylation in response to environmental stress conditions were described in rice [304,305], maize [306], foxtail millet [307], and sesame [308]. These and additional surveys of comparative DNA methylation patterns in response to environmental conditions reveal many possible targets for which causality should be assessed. 

While precise and highly efficient targeted methylation/demethylation can be achieved in *Arabidopsis*, the efficiency and applicability of these tools are yet to be demonstrated in crop plants. Moreover, additional limitations hinder the use of DNA methylation editing for crop epi-breeding. First, the occurrence of genome-wide off-target methylation (including chloroplast methylation) was reported in the *Arabidopsis* system, which could be reduced to background levels when modifying the components of the expression cassettes [198,296], implying the importance of an in-depth calibration of the system for each plant species. Furthermore, the choice of the effector protein will potentially affect targeting efficacy, so additional calibration is needed to identify the best component(s). Importantly, DNA methylation is part of a complex transcriptional regulatory system, and the sole modification of DNA methylation patterns may not be sufficient to affect gene transcription. In this respect, editing of histone tail modifications is yet to be explored. Considering the dynamic nature of epigenetic marks, the transgenerational stability is of central importance. Although short-term heritability of DNA methylation patterns was well demonstrated in several species, including rice and wheat [309,310], most studies are limited to one or two generations, so the long-term methylation pattern stability, if desired, remains to be studied. Additionally, the level of stability/inheritance might be dependent on the genetic context and histone marks occupancy [300,311]. It should be noted, however, that in *Arabidopsis*, selected epi-alleles were found to be stable for more than twenty generations [300]. Epigenetic editing technologies continue to develop, providing insights into the mechanism of editing and transgenerational stability. Thus, more precise and stable modifications are expected, as was recently reported in a mammalian cell line system [312].

Targeted changes in DNA methylation can therefore be exploited to modulate gene expression and create novel epialleles, including gain of function epi-alleles, which are less likely to be achieved using the current genetic editing methods. Furthermore, these tools could be employed to repair undesired methylation changes arising, for instance, from plant regeneration through tissue culture [313] or by stressful environmental conditions in a previous generation [185]. The induced methylation could be heritable in the absence of the initial activating construct, making targeted manipulation of DNA methylation an approach to permanently modify a locus [190]. Importantly, the components needed to manipulate DNA methylation can be delivered through direct transfer of RNA molecules and proteins to plant cells [314,315], circumventing the need for genetic transformation and paving the way to the manipulation of DNA methylation in various crop species.

## 5. Conclusions

As described in this review, it is now well established that epigenetic mechanisms control phenotype variations, agronomic traits, and particularly responses to environmental fluctuations, notably through priming (Figure 1). Therefore, epigenetic-knowledge-based technologies provide a promising toolbox for breeders. Indeed, epigenetic diversity (natural or induced by different ways) can be exploited as a source of phenotypic diversity or priming, for the creation of new varieties acclimated to climate changes, or as molecular epi-markers for selecting elite individuals or for the identification of new candidate genes. The transfer of the knowledge on epigenetic signatures associated with stress tolerance/resistance in crops is still at the beginning. There are typically hundreds of molecular markers already mapped in crop genomes associated with important traits, including tolerance to environmental stresses. On the other hand, few epigenetic markers dealing with agronomic traits have been mapped and used in breeding [316]. While the potential applications of epigenetic-based strategies in crops have already been reported [119,182,246,317,318], this review not only provides an updated overview of the rapidly evolving field, but also discusses novel strategies as well as challenges on our way to engineer crop improvement (Figure 1). The main challenges to reach crop improvement using epigenetics could be summarised as follows:

- Identification of new epigenetically regulated traits

The elucidation of epigenetic variability and molecular mechanisms involved in plant adaptation to climate change will assist breeders in developing broad-resistant and plastic cultivars obtained from the phenotypic variability currently available.

- Facilitate the selection of elite genotypes for the development of new cultivars/varieties

The identification of transgenerational epigenetic modifications will allow one to increase the number of molecular markers mapped in the genome, increasing the association of markers with important agronomic traits. This will facilitate the improvement of the selection of more favourable climate-smart crop varieties that are more resilient to climate change.

- Understand how epigenetic mechanisms trigger resistance/tolerance to multiple stresses and evaluate their stability

Better knowledge of the molecular epigenetic mechanisms underlying crop responses to environmental stresses will allow breeders to develop cultivars with higher yield stability during seasons characterised by different climates. It is needed to clarify the role of thousands of different spliced transcript variants in the regulation of expression of genes involved in key agronomic traits. More research and techniques are needed to gain insight into the function of this large part of untranslated RNA. The connections between these transcripts and epigenetic modifications of hereditary material (DNA methylation and chromatin modifications) needs to be clarified. In addition, the identification of genes (such as *DDM1*) modulating DNA methylation activity through orthology between closely phylogenetic plant species will be essential in order to map epigenetic marks involved in de novo or maintaining methylation linked not only to genomic imprinting but also transgenerational memory of important agronomic traits [155]. 

Another important issue is represented by the requirement of identifying epigenetic modifications that are stable and inherited to the filial generation, distinguishing them from those transiently induced. Indeed, epigenetic modifications are able to modify the activity of TEs, notably in stressful environments. These TEs, by inserting at new genomic positions, induce stable genetic (and epigenetic) variations. Recently, Baduel and Colot [42] have argued that “TE-associated epivariation, whether stable or not, lies predominantly in its capacity to modulate TE mobilization in response to the environment, hence providing hard-wired opportunities for the flexible exploration of the phenotypic space”. The ability to induce transposon bursts in crops, as already shown by Thieme et al. [319], is also a promising tool for breeders. 

Among the inherited ones, another challenge is to identify those that are inter-generational (transmitted only for a few generations and then eliminated) from those that are trans-generational (transmitted for many generations and responsible for stress memory). Further investigation is needed to identify epi-alleles which are stable during breeding. Another important issue will be to identify epigenetic changes that are stable during successive generations in order to render efficient selection during the breeding. 

Finally, recent findings focused on the role of 21-nt phasiRNAs in epigenetic modulation of chromatin re-structuring highlight the importance of mapping such epigenetic marks in the genome sequence of key crops. This work will allow the identification of key epigenetic players involved in imprinting and crop adaptation for the development of epigenetic markers that can be utilised for crop genetic improvement. In addition, small RNAs and cross-kingdom RNA interference are part of plant immune responses against pathogens [318]. Thus, pathogens and pests can be controlled by sRNAs, targeting their essential or pathogenicity genes, raising the possibility of plants that are protected from diseases by a novel, eco-friendly, durable, and highly specific RNA fungicide or pesticide [320,321].

- Improve integrative approaches, statistics, and modelling for crops using epigenetics

The integrative statistical analysis of “-omics” data increases study power and provides insight into the interactions between the various mechanisms of molecular regulation, such as epigenetic determination of the phenotype. The success of the trans-omics approach (reconstruction of molecular networks by connecting multiple -omic data) will be associated with the development of dedicated tools using mathematical network models or kernels and kernel-based methods for crop improvement [66]. Recently, Champigny [322] applied statistical learning experiments to genetically diverse populations of *Populus balsamifera* trees grown at two common garden sites and showed that traits in novel genotypes can be modelled using small numbers of methylated DNA predictors. The authors proposed that DNA-methylation-based models can be used as a strategy to validate the identity, prevalence, or quality of agroforestry products. Development of mathematical models will allow the prediction of the identification of inherited epigenetic phenotypes which could be important for breeding programs. These models may be used in the study of epigenetic mark transmissibility, to integrate them in genome-wide association analysis [323]. The perspectives for crop applications should be envisaged in the near future.

- Reduce loss of genetic variability

The characterisation, maintenance, and increase in epigenetic variation will promote total genetic variance which could be exploited in the genetic breeding. This will compensate for the loss of genetic variability due to the use of a limited number of cultivars for each crop.

- Use epigenetics and priming for enhanced management of a/biotic stresses in crops

Epigenetic analysis will enable the discovery of new epi-alleles involved in tolerance/resistance to those biotic stresses regulated by single genes. For example, in rice, a high number of epimutations were shown to be involved in drought tolerance. These altered methylation markers were trans-generationally inherited. They were shown to play a role in the tillering and grain-filling stage [45]. Epigenetic analysis will also allow us to clarify mechanisms of stress-priming to enhance a crop’s ability to tolerate environmental stress without any reduction in yield and yield components. Another important challenge is the understanding and the characterisation of a different epigenetic status of primed/unprimed seeds. In addition, seed banking and conservation are performed at low temperatures, to maintain a dormant state until planted, and could lead to significant yield loss. Recent data suggest that epigenetic mechanisms may play a key role in, and should be assessed to improve, seed storage practices [324,325].

- Reduce efforts on molecular breeding

The standardisation and normalisation of epigenetic pipelines, methods used in both lab and in silico analysis, will allow the development of methodologies and approaches which could be used by breeders for molecular-marker-assisted selection. Epi-genotyping will allow us to identify epi-alleles from genetic ones for the discovery of spontaneous epi-alleles. The development of epi-mutagenesis and epigenome editing will allow the engineering of the crop epigenome. In addition, recent advances in single-cell sequencing technology concerning genomics and epigenomics are promising for dissecting cell heterogeneity [326]. This could be useful to support crossover detection in gametophyte or in vitro technologies based on cell regeneration. Finally, the identification of epigenetic variants involved in crossover repression will be helpful to promote linkage and maintain association between preferred agronomic traits. More generally, changes to epigenetic marks can affect the rate and location of crossovers and may represent a valuable tool for breeders [83]. However, further research is still necessary to identify the suitable strategy for a given crop. For example, next-generation sequencing will be useful to identify gene alleles encoding histone variants positively linked with increased crossover events in order to enhance genetic variation created by artificial crossing activities.

- Clarify epigenetic mechanisms for public acceptance

The clarification of meaning, nature, and methodologies in epigenetics will allow public opinion to understand the differences between transgenic and genome editing technologies, increasing the value of any epigenetic discovery and highlighting the role of environmental and natural changes on the genetic material. This will allow us to explain how safe and healthy these approaches are in comparison with other traditional and molecular breeding methods.

- Requirement for further research in plant epigenetics and synergy between academic and private or public partners.

For this reason, the EPI-CATCH consortium has been launched in the framework of a recently funded COST Action with the aim of studying the epigenetic adaptation to climate change. This project will be important for strengthening the network between academic and private research in this under-investigated field in plant sciences. The EPI-CATCH COST Action (2020–2024) involves more than 25 countries from Europe and nearby COST-associated countries and aims to develop a consortium to address the challenges of climate changes at the epigenetic level. 

**Table 1:** Examples of epigenetics for crop improvement.

Species	Topic	Epigenetic Modification	Main Conclusions towards Crop Improvement	Reference
*Arabidopsis*, rice, maize, and other plants	Identification of a sexual-lineage-specific DNA methylation signatures occurred by RNA-directed DNA methylation (RdDM) during plant gametogenesis.	DNA methylation	The clarification of genes and oligonucleotides involved in the modulation of RNA-directed DNA methylation and their mapping in sequence genomes will be of extreme interest to develop new molecular markers associated with fertility, male sterility, and self-incompatibility.	[327]
*Arabidopsis*, tomato	Rootstock epigenetic variation in a comparative analysis in *Arabidopsis* and tomato.	Small RNA	They showed how the enhanced plant vigour phenotypes of the *MSH1* system is reproducible in tomato field size experiments and therefore demonstrated how epigenetic perturbation strategies can be used in crops.	[20]
*Arabidopsis*, white clover	Diminishing the differences between memorised and wild-type plants by DNA demethylating chemical.	DNA methylation	Studies focused on description of DNA methylation in stress memory phenomenon.	[141]
Basket willow, spinach, *Arabidopsis*	More frequent flowering after treatment by DNA demethylating chemical.	DNA methylation	Artificial induction of flowering.	[131]
Canola	In an isogenic canola population, the authors showed how energy use efficiency can be selected artificially through an epigenetic feature to increase yield in hybrids.	DNA methylation and histone modifications	The shaping of the epigenome has the potential to artificially increase yield in crops.	[231]
Cork oak	Interplay between epigenetic markers related to the acclimation of cork oak plants to high temperatures.	DNA methylation and histone modifications	Increased DNA methylation under high temperature. Dynamics of methylation/demethylation patterns over stress. DNA methylation and histone H3 acetylation have opposite effects and a particular dynamic.	[328]
Cotton	Epigenomic and functional analyses reveal roles of epialleles in the loss of photoperiod sensitivity during domestication of allotetraploid cottons.	DNA methylation	DNA methylation is suggested to affect photoperiodic flowering time and seed dormancy.	[303]
Grapevine	Epigenetic memory induced by stress.	DNA methylation	Conservation of DNA methylation changes in response to medium-high temperatures in regenerated plants.	[195]
Grapevine, poplar	Locally established unique epigenetic marks used for authentication/declaration of origin.	DNA methylation	Authentication of plant origin; use of locally adapted clones.	[203]
Grapevine, fruit, woody- crop, and forest trees	Woody plants grafting and epigenetic changes.	All chromatin interactions	Woody species grafting is a promising agriculture technology for generating improved woody plants that can face environmental challenges without major compromise in yield and quality and with low input requirements.	[14]
Madagascar periwinkle	Production of medicinal secondary metabolites.	DNA methylation	An epigenetic regulation of specialised metabolisms (alkaloids) was unravelled in C roseus, notably targeting transcription factors, which in turn may control the expression of enzyme-encoding genes. This could be exploited to improve the production of secondary metabolites for pharmaceutical applications using plant biotechnologies.	[71]
Maize	Defence priming to herbivores.	DNA methylation	Possibility to increase plant defence by application of volatiles related to this mechanism.	[329]
Maize	The maize methylome influences mRNA splice sites and reveals widespread paramutation-like switches guided by small RNA.	DNA methylation	The methylation map will provide an invaluable resource for epigenetic studies in maize and how methylation patterns can be used to predict key phenotypes.	[240]
Maize	DNA methylation variation (and specific DMRs) as a powerful phenotypic predictor, independent of genetic polymorphism data.	DNA methylation	A first effort to perform genome-wide association analysis using epigenetic data in a crop species.	[78]
Maize	Analysis of DNA methylation in different growth zones of maize leaves and transcriptional analysis of genes involved in chromatin remodeling, cell cycle progression, and growth regulation.	DNA methylation	DNA methylation controls cell division in maize leaves and correlates with the mitotic exit and entering cell expansion.	[28]
Maize	Investigation of the diversity of DNA methylation states and their association to genotype and gene expression in maize inbred lines.	DNA methylation	Many genes located near the identified DMRs have tissue-specific expression. The expression patterns of over 300 of these genes strongly correlate with the methylation state and are often stably inherited.	[70]
Maize	H3K4me3 and H3K27me3 changes involved in the memory of drought stress. Floral patterning is affected in response to stress as a possible consequence of epigenetic changes.	Histone modifications	Coordinated transcriptomic and epigenomic reprogramming of maize plants in response to a main abiotic stress with an impact on plant development and recovery to the stress. Identification of different types of memory genes that may be used as future targets to enhance plant resilience to stress. Identification of putative stress marks which are not associated to direct transcriptional changes.	[192]
Maize	Parental divergence in sRNA are strong predictors for grain yield in the hybrids.	Small RNA	Epigenetic measurements may be used as complementary biomarkers in crops.	[220]
Maize, rice	Epigenetic mechanisms involved in meiotic events during pollen development.	Non-coding mechanisms	Mapping of genes encoding 21-nt phasiRNAs will allow the development of epi-molecular markers usable for the selection of genotypes with different rates of occurrence of meiotic events.	[84]
Maize, wheat, barley, rice, chickpea, pea, tomato	Possible applications of epigenetics in climate-smart crop breeding.	DNA methylation and chromatin modifications	Gaining insight into epigenetic mechanisms will allow improvement of crop adaptation and resilience to environmental stresses, producing a next generation of stable climate-smart crops.	[182]
Oil palm	Epiallele responsible for poor fruit production in oil palm.	DNA methylation and small RNA	The ability to predict and cull mantling at the plantlet stage will facilitate the introduction of higher-performing clones and optimise environmentally sensitive land resources.	[108]
Poplar	Memory of drought stress in cultivated trees.	DNA methylation	Epigenetic memory in the meristem of stressful environmental conditions occurred during the preceding summer period. This memory may facilitate tree acclimation through priming for cuttings. Clonal propagation of primed trees.	[65]
Poplar	Drought tolerance in trees.	DNA methylation	RNAi-*ddm1* lines are more tolerant to drought stress. DNA methylation controls hormonal pathway genes (salicylic acid, cytokinins, ethylene) and some transcription factors, but also the activation of TEs that induce mutations potentially near or in genes. This, taking place in the shoot apical meristem, may be transmitted mitotically to primed organs and to the next generation. Confirmation is needed.	[155]
Rapeseed, white oak	DNA hypomethylation characterises somatic embryogenesis initiation in quercus trees.	DNA methylation	DNA hypomethylation characterises somatic embryogenesis initiation in clonal propagation techniques of forest trees	[280,285]
Rapeseed	DNA methylation changes during pollen development and cell reprogramming in somatic embryogenesis.	DNA methylation	DNA hypomethylation is required for plant cell reprogramming to initiate microspore embryogenesis and doubled haploid production for crop breeding.	[96]
Rapeseed, barley	Epigenetic modulators that reduce DNA methylation promote cell reprogramming and microspore embryogenesis for double haploid production.	DNA methylation	DNA de-methylating agents promote cell reprogramming in microspore embryogenesis and doubled-haploid production, favouring acceleration of crop breeding programs.	[128]
Rapeseed, barley	Small molecules that produce H3K9 de-methylation to promote cell reprogramming and somatic embryogenesis in crop species.	Histone modifications	Novel small molecules that decrease histone H3K9 methylation levels promote cell reprogramming in microspore embryogenesis and doubled haploid production, favouring acceleration of crop breeding programs.	[284]
Rice	Long-term semantic memory to salinity stress.	DNA methylation	Rice is considered a salt-sensitive crop; molecular processes involved in memory to stress may help to breed more resistant plants.	[172]
Rice	Phasing analysis of the transcriptome and epigenome in a rice hybrid.	DNA methylation	Developed a phasing pipeline that provides insights into alternative splicing, interaction networks, trans-acting regulation, and the inheritance of DNA methylation in rice.	[40]
Rice	Exploring the role of DNA methylation variations in rice adaptation to drought stress.	DNA methylation	Multi-generational drought improves drought adaptability of offspring, which could be linked to non-random appearance of drought-induced transgenerational epimutations. Some of the genes related to these epimutations are directly involved in stress-responsive pathways.	[45]
Rice	Identification of DNA methylation transgenerational inherited changes in heavy-metal-responsive genes.	DNA methylation	How plants can cope better with heavy metal stress through heritable changes in DNA methylation.	[193]
Rice	A large-scale whole-genome sequencing analysis to assess the specificity of genome editing by Cas9 and Cpf1 nucleases in rice.	Whole-genome sequencing	Cas9 and Cpf1 nucleases are very specific in generating targeted DNA modifications, and off-targeting can be avoided by designing guide RNAs with high specificity.	[51]
Rice	DNA methylation and H3K9me2 was shown to repress plant crossover hotspots.	DNA methylation and chromatin modifications	Important implications in the creation of genetic variability produced by breeding activities, because it allows better selection of parental genotypes usable for artificial crossings.	[89]
Rice, pea, tomato	Controlled recombination through counting on crossovers can facilitate plant breeding.	Epigenetic modifications and crossovers	Use of genome editing reagents that induce double-stranded breaks (DSBs) or modify the epigenome at desired sites of recombination, and manipulation of cofactors, are increasingly applicable approaches for achieving this goal. These strategies for ‘controlled recombination’ have potential to reduce the time and expense associated with traditional breeding, reveal currently inaccessible genetic diversity, and increase control over the inheritance of preferred haplotypes.	[83]
Rubber trees	Chilling-induced DNA demethylation is associated with the cold tolerance of *Hevea brasiliensis*	DNA methylation	Chilling treatments induced methylation changes and transcriptional activity of methylation and cold-stress-related genes.	[51]
Soybean	DNA methylation reprogramming during soybean seed development.	DNA methylation	DNA methylation dynamically changes during soybean seed maturation, affecting the expression of multiple genes. Majority of the DMR genes in the CHH context are downregulated, and closely linked to DNA replication and cell division. This seems to be a protective mechanism that keeps transposons silent to prevent inactivation of genes essential for seed development.	[31]
Soybean	DNA methylation patterns in soybean root hairs.	DNA methylation	DMRs in each methylation context have distinct methylation patterns between root hairs and stripped roots, and under heat stress. At normal temperature, root hairs are more hypermethylated than stripped roots. Upon heat stress, both cell types are hypomethylated in each context, especially in the CHH context.	[59]
Soybean	DNA methylation and histone modifications of salt-responsive transcription factor genes.	DNA methylation and histone modifications	Salinity stress was shown to affect the methylation status of several transcription factors (one MYB, one b-ZIP, and two AP2/DREB family members). For some of them, DNA methylated transcription factors were correlated with an increased level of histone H3K4 trimethylation and H3K9 acetylation, and/or a reduced level of H3K9 demethylation in various parts of the promoter or coding regions.	[330]
Sugar beet	Tolerance to bolting.	DNA methylation	Tolerance to bolting is an agronomic trait for biennial cultivated sugar beet. Bolting is associated with the use of sucrose root stock and should be avoided in the field. Here, tolerance to bolting was correlated to epigenomic polymorphism in DNA methylation, notably in genes involved in cold acclimation, hormonal pathway genes, and flowering genes.	[63,242]
Tobacco	Abiotic stress induces demethylation and transcriptional activation of a gene encoding a glycerophosphodiesterase-like protein in tobacco plants.	DNA methylation	Aluminum stress, salt, and low temperature treatments induced demethylation patterns. These results suggested a close correlation between methylation and expression of NtGPDL upon abiotic stresses with a cause–effect relationship.	[331]
Tobacco, potato	Reactivation of silenced transgenes by DNA demethylating chemicals.	DNA methylation	More efficient genetic transformation of plants.	[137]
Tobacco, rapeseed onion, barley, cork oat	Method to evaluate global DNA methylation changes and nuclear pattern distribution in a variety of crop and forest species.	DNA methylation	Method to estimate differences in global DNA methylation levels among different cell types and organs during development, which can help to evaluate epigenetic reprogramming events associated with plant growth and adaptation.	[281,282]
Tomato	Epigenetic marks in an adaptive water stress-responsive gene in tomato roots under normal and drought conditions.	DNA methylation	Drought induces the removal of methyl marks in the regulatory region (at 77 of the 142 CNN sites) DNA methylation involved in drought acclimation.	[332]
Tomato	A DEMETER-like DNA demethylase governs tomato fruit ripening.	DNA methylation	Active DNA demethylation is central to the control of ripening in tomato. RNAi SlDML2 knockdown results in ripening inhibition via hypermethylation and repression of the expression of genes encoding ripening transcription factors and rate-limiting enzymes of key biochemical processes such as carotenoid synthesis.	[75]
Tomato	Chilling-induced tomato flavor loss is associated with altered volatile synthesis and transient changes in DNA methylation.	DNA methylation	Changes in DNA methylation are associated with reduced levels of specific volatiles and reductions in transcripts encoding key volatile synthesis enzymes during fruit ripening. RNAs encoding transcription factors essential for ripening, including RIPENING INHIBITOR (RIN), NONRIPENING, and COLORLESS NONRIPENING, are reduced in response to chilling and may be responsible for reduced transcript levels in many downstream genes during chilling. Those reductions are accompanied by major changes in the methylation status of promoters.	[333]
Tomato	Single-base resolution methylomes of tomato fruit development reveal epigenome modifications associated with ripening.	DNA methylation	DNA methylation changes through fruit ripening: the epigenome is not static during development and may have been selected to ensure the fidelity of developmental processes, such as ripening.	[334]
Tomato	Relationships between genome methylation, levels of non-coding RNAs, mRNAs, and metabolites in ripening tomato fruit.	DNA methylation	Multiple changes in gene methylation were linked to the ethylene pathway and ripening processes.	[335]
Tomato	Naturally occurring epialleles determine vitamin E accumulation in tomato fruits.	DNA methylation	Vitamin E content is controlled by mQTL9-2-6—an expression QTL associated with differential methylation of a SINE retrotransposon located in the promoter region of VTE3—that catalyses one of the final steps in the biosynthesis of vitamin E. These findings indicate, therefore, that naturally occurring epialleles are responsible for regulation of a nutritionally important metabolic QTL.	[115]
Wheat	The contribution of epigenetic modifications to the expression divergence of three TaEXPA1 homoeologs in hexaploid heat.	DNA methylation and chromatin modifications	Epigenetic modifications contribute to the expression divergence of three TaEXPA1 homoeologs during wheat development.	[30]

## Data Availability

Not applicable.
